# Novel Mutations in the Asparagine Synthetase Gene (*ASNS*) Associated With Microcephaly

**DOI:** 10.3389/fgene.2018.00245

**Published:** 2018-07-13

**Authors:** Dorit Schleinitz, Anna Seidel, Ruth Stassart, Jürgen Klammt, Petra G. Hirrlinger, Ulrike Winkler, Susanne Köhler, John T. Heiker, Ria Schönauer, Joanna Bialek, Knut Krohn, Katrin Hoffmann, Peter Kovacs, Johannes Hirrlinger

**Affiliations:** ^1^IFB AdiposityDiseases, Leipzig University Medical Center, University of Leipzig, Leipzig, Germany; ^2^Division of Nephrology, Department of Internal Medicine, Leipzig University Medical Center, University of Leipzig, Leipzig, Germany; ^3^Division of Neuropathology, Department of Diagnostic, Leipzig University Medical Center, University of Leipzig, Leipzig, Germany; ^4^Hospital for Children and Adolescents, Faculty of Medicine, University of Leipzig, Leipzig, Germany; ^5^Medizinisch-Experimentelles Zentrum, Faculty of Medicine, University of Leipzig, Leipzig, Germany; ^6^Carl-Ludwig-Institute for Physiology, Faculty of Medicine, University of Leipzig, Leipzig, Germany; ^7^Institute of Biochemistry, Faculty of Life Sciences, University of Leipzig, Leipzig, Germany; ^8^Institute of Human Genetics, Martin Luther University Halle-Wittenberg, Halle, Germany; ^9^Core Unit DNA Technologien, Faculty of Medicine, University of Leipzig, Leipzig, Germany; ^10^Department of Neurogenetics, Max-Planck-Institute for Experimental Medicine, Göttingen, Germany

**Keywords:** microcephaly, asparagine synthetase, mutation, compound heterozygous, exome sequencing, genetic variants

## Abstract

Microcephaly is a devastating condition defined by a small head and small brain compared to the age- and sex-matched population. Mutations in a number of different genes causative for microcephaly have been identified, e.g., *MCPH1, WDR62*, and *ASPM*. Recently, mutations in the gene encoding the enzyme asparagine synthetase (*ASNS*) were associated to microcephaly and so far 24 different mutations in *ASNS* causing microcephaly have been described. In a family with two affected girls, we identified novel compound heterozygous variants in *ASNS* (c.1165G > C, p.E389Q and c.601delA, p.M201Wfs^∗^28). The first mutation (E389Q) is a missense mutation resulting in the replacement of a glutamate residue evolutionary conserved from *Escherichia coli* to *Homo sapiens* by glutamine. Protein modeling based on the known crystal structure of ASNS of *E. coli* predicted a destabilization of the protein by E389Q. The second mutation (p.M201Wfs^∗^28) results in a premature stop codon after amino acid 227, thereby truncating more than half of the protein. The novel variants expand the growing list of microcephaly causing mutations in *ASNS*.

## Background

Microcephaly is a devastating condition defined by a small head compared to the age- and sex-matched population, often defined as a head circumference more than three standard deviations below the age- and sex-matched means. In most cases, microcephaly (“small head") is associated with microencephaly (“small brain”; Gilmore and Walsh, 2013). Children with microcephaly often have impaired cognitive development, facial distortions, hyperactivity, seizures, and other brain and neurological impairments. While some patients only show rather mild disabilities, other patients are severely affected and require lifelong intensive care. Both, genetic as well as acquired causes have been described for congenital microcephaly and several genes have been identified causing autosomal recessive primary microcephaly (Faheem et al., 2015). These genes mostly affect mitosis of neural progenitors, resulting in reduced numbers of neurons. Furthermore, mutations in other genes have been associated with microcephaly, which affect cellular processes other than neuronal development (Morris-Rosendahl and Kaindl, 2015).

Originally in 2013, three causative mutations in the gene coding for asparagine synthetase (*ASNS*) have been identified in patients suffering from microcephaly in four families (Ruzzo et al., 2013; ASNS deficiency, ASNSD; OMIM #615574). Since then several mutations were reported in patients with microcephaly in scientific publications (Ruzzo et al., 2013; Alfadhel et al., 2015; Ben-Salem et al., 2015; Palmer et al., 2015; Gataullina et al., 2016; Seidahmed et al., 2016; Gupta et al., 2017; Sacharow et al., 2017; Sun et al., 2017; Yamamoto et al., 2017; Abhyankar et al., 2018; Galada et al., 2018; for an overview see Gupta et al., 2017; Lomelino et al., 2017 and **Table 1**) and three others have been reported in poster form or in the internet (**Table 1**) from almost all around the world.

**Table 1:** New and published *ASNS* genetic variants associated with microcephaly.

No.	Position in coding sequence NM_133436.3	Position in protein sequence NP_597680.2	Published by (→ comments)	dbSNP entry for the base position in coding sequence NM_133436.3 [ClinVar:clinical significance]
1	c.17C > A	p.A6E	Ruzzo et al. (2013) *Neuron* 80, 429–441	rs398122975 C > A (p.A6E) [pathogenic/likely pathogenic] and C > T (p.A6V)
2	c.146G > A	p.R49Q	Sacharow et al. (2017) *Molecular Genetics and Metabolism* 123, 317–325	rs769236847 G > A (p.R49Q) [likely pathogenic]
3	c.198_202delATATC	p.K66Nfs^∗^10	Reed (2016) Poster at Annual Clinical Genetics Meeting	
4	c.224A > G	p.N75S	Galada et al. (2018) Congenital Anomalies (Kyoto)	rs747624770 A > G (p.N75S)
5	c.413A > C	p.D138A	Galada et al. (2018) Congenital Anomalies (Kyoto)	rs797045306 A > T (p.D138V) [pathogenic]
6	c.434T > C	p.L145S	Yamamoto et al. (2017) *Brain & Development* 39, 236–242	
7	c.601delA	p.M201Wfs^∗^28	This publication	
8	c.728T > C	p.V243A	Abhyankar et al. (2018) *Clinical Case Reports* 6, 200–205	rs148111963 T > C (p.V243A) [likely pathogenic]
9	c.740T > G	p.L247W	Yamamoto et al. (2017) *Brain & Development* 39, 236–242	
10	c.866G > C	p.G289A	Palmer et al. (2015) *Molecular Genetics and Metabolism* 116, 178–186	rs369633015 G > A (p.G289D)
11	c.1010C > T	p.T337I	Palmer et al. (2015) *Molecular Genetics and Metabolism* 116, 178–186	
12	c.1019G > A	p.R340H	Sun et al. (2017) *JIMD Reports* 34, 1–9	
13	c.1084T > G	p.F362V	Ruzzo et al. (2013) *Neuron* 80, 429–441	rs398122973 T > G (p.F362V) [pathogenic]
14	c.1097G > A	p.G366E	Abhyankar et al. (2018) *Clinical Case Reports* 6, 200–205	
15	c.1138G > T	p.A380S	Gupta et al. (2017) *Metabolic Brain Disease* 32, 1889–1900	rs758183057 G > C (p.A380P)
16	c.1165G > C	p.E389Q	This publication	rs948326794 G > T (p.E389^∗^)
17	c.1193A > G	p.Y398C	Alfadhel et al. (2015) *JIMD Reports* 22, 11–16 → Variant reported as c.1160A > G (p.Y377C)	rs773348232 -/T (p.Y398Lfs^∗^18) [likely pathogenic]
	c.1193A > G	p.Y398C	Ben-Salem et al. (2015) *Metabolic Brain Disease* 30, 687–694	
	c.1193A > G	p.Y398C	Seidahmed et al. (2016) *BMC Neurology* 16, 105 → Variant reported as c.944A > G (p.Y315C) as numbered according to sequence NM_001178076.1/ENST00000455086.5	
18	c.1211G > A	p.R404H	Galada et al. (2018) *Congenital Anomalies (Kyoto)*	rs774808316 G > A (p.R404H)
19	c.1219C > T	p.R407^∗^	Seidahmed et al. (2016) *BMC Neurology* 16, 105 → Given in two versions (c.970C > T/p.R324^∗^; c.1219C > T/p.R407^∗^), as reference sequences are mixed (NM_0011780761.1; NM_133436.3)	rs1140424 C > T (p.R407^∗^)
20#	c.1279-1281[TCC]	p.S427P	http://thejoyofjules. blogspot.de/2016/12/ genetics-results-asparagine-synthetase. html → Self-reported	3 SNPs, one for every position in the codon: c.1279 rs1057518341 T > C (p.S427P) (likely the mutation) [likely pathogenic] c.1280 rs753038843 C > T (p.S427F) c.1281 rs768750423 C > T (p.S427S)
21	c.1439C > T	p.S480F	Gataullina et al. (2016) *Neuropediatrics* 47, 399–403	rs754043007 C > T (p.S480F) [likely pathogenic]
22	c.1466T > A	p.V489D	Yamamoto et al. (2017) *Brain & Development* 39, 236–242	
23#	c.1555-1557[CGT]	p.R519H	http://thejoyofjules. blogspot.de/2016/12/ genetics-results-asparagine-synthetase. html → Self-reported	2 SNPs, first and second position of the codon: c.15555 COSMIC database COSM1093405 c.15555 C > T (p.R519C) c.1556 rs568570377 G > A (p.R519H) (likely the mutation) [likely pathogenic]
24	c.1623_1624delGA	p.W541Cfs^∗^5	Yamamoto et al. (2017) *Brain & Development* 39, 236–242	c.1624 rs755055878 A > T (p.I542F)
25	c.1648C > T	p.R550C	Gataullina et al. (2016) *Neuropediatrics* 47, 399–403	rs398122974 C > T (p.R550C) [pathogenic/likely pathogenic]
	c.1648C > T	p.R550C	Ruzzo et al. (2013) *Neuron* 80, 429–441	
26	c.1649G > A	p.R550H	Galada et al. (2018) *Congenital Anomalies (Kyoto)*	rs552452349 G > A (p.R550H)

*Variants are given in the order of their occurrence in the coding sequence. ^#^Amino acid change reported in an online blog without annotation of the coding sequence position and change. NCBI:ClinVar – aggregates information about genomic variation and its relationship to human health (Landrum et al., 2016).*

Asparagine synthetase is a metabolic enzyme (EC 6.3.5.4) catalyzing the reaction L-aspartate + L-glutamine + ATP + H_2_O →L-asparagine + L-glutamate + AMP + PPi (Horowitz and Meister, 1972). ASNS is expressed almost ubiquitously with higher expression levels in brain but very low levels in liver (Ruzzo et al., 2013; Lomelino et al., 2017). Consequently, it has been proposed that insufficient supply of asparagine within the brain underlies brain malformation and malfunction in ASNSD (Ruzzo et al., 2013). As ASNS metabolically connects the four amino acids L-aspartate, L-asparagine, L-glutamate, and L-glutamine also a dysregulation of the balance of these amino acids in the brain might contribute to the pathophysiology of ASNSD (Lomelino et al., 2017; Sacharow et al., 2017). ASNS has been intensively studied in cancer research as tumors capable to synthesize asparagine *de novo* via ASNS may display resistance to the treatment by asparaginase (Horowitz et al., 1968; Aslanian et al., 2001; Richards and Kilberg, 2006), and ASNS expression in human solid tumors was correlated with survival prognosis (Zhang et al., 2013; Panosyan et al., 2016). Furthermore, glutamine-dependent asparagine synthesis via ASNS is indispensable for endothelial cell growth which is important for angiogenesis supporting invasive tumor growth and metastasis (Folkman, 2002; Huang et al., 2017). Consistently, in fibroblasts derived from patients with ASNSD proliferation was markedly reduced under conditions of asparagine deprivation (Palmer et al., 2015; Sacharow et al., 2017). However, while the association of ASNS mutations and microcephaly is now well-established, the pathophysiology of these mutations is still unclear.

We here report two novel mutations in *ASNS* in a German family with two girls suffering from microcephaly, which are inherited in a compound heterozygous manner in the family.

## Compliance With Ethical Standards

All procedures performed in studies involving human participants were in accordance with the ethical standards of the institutional and/or national research committee and with the 1964 Helsinki declaration and its later amendments or comparable ethical standards. Informed written consent was obtained from all individual participants or their legal representatives (parents) included in the study. The study was approved by the ethical committee of the University of Halle.

## Case Presentation

**FIGURE 1:**
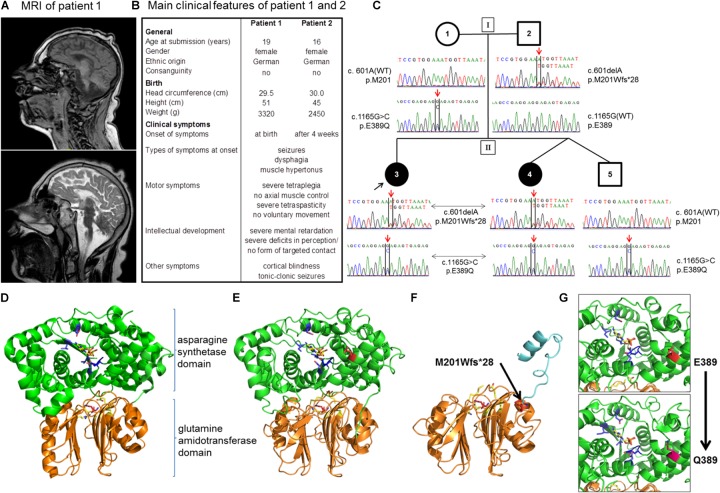
Patients and novel mutations in the *ASNS* gene. **(A)** Sagittal MRI images (upper image: T1-weighted, lower-image: T2-weighted) from patient 1 at the age of 10 years demonstrating a severe microcephaly with a pronounced cortical and subcortical atrophy of the cerebral hemispheres. **(B)** Overview of the main clinical features of patients 1 and 2. Note that disease symptoms are nearly identical in the two patients except for a slightly later onset of the first neurological symptoms in patient 2. **(C)** Pedigree of the family. Below the symbols the sequencing results of Sanger sequencing spanning the sequence around c.601 and c.1165 for each member of the family are shown. **(D–G)** Localization of disease mutations in a model of the human asparagine synthetase protein. The human asparagine synthetase protein structure **(E,F)** were modeled using the asparagine synthetase B crystal structure from *E. coli* (pdb id: 1CT9) as a template **(D)**. **(D)** Structure of *E. coli* asparagine synthetase B. *Orange*/*lower part* of the molecule: glutamine amidotransferase domain with glutamine (*purple*) in the binding pocket (important residues in *yellow*); *green*/*upper part* of the molecule: asparagine synthetase domain with AMP (*yellow*) in the binding pocket (important residues in *blue*). **(E)** Human asparagine synthetase model visualized until residue 536. The ASNS C-terminus is very flexible and not resolved in the crystal structure of *E. coli* ASNS B. Important residues for glutamine binding (R49, N75, E77, and D97 in *yellow*) and glutamine (*purple*) in the binding pocket are shown in the glutamine amidotransferase domain (conserved). E389 (presented in *red*) is the residue for which the heterozygous mutation E389Q was found in the family. **(F)** Modeling of the M201Wfs^∗^28 mutation (*red, black arrow*) illustrates the mutant protein lacking the asparagine synthetase domain due to the premature stop codon. **(G)** Detailed view of the human asparagine synthetase domain with residues E389 (*red*; *upper part*) and Q389 (*pink*; *lower part*).

The two affected sisters were born to healthy non-consanguineous German parents with unremarkable family history. Patient 1 was a full-term born female baby with a weight of 3,320 g (50th percentile) and a height of 51 cm (10th percentile). The head circumference at birth measured 29.5 cm, corresponding to a circumference below the 3rd percentile in female neonates. Of note, a microcephaly was already observed in routine ultrasonography during pregnancy. Until the age of 2 years, the head circumference was determined in short time intervals demonstrating an increase in size that parallels the standard growth curves with respective to the gradient angle in the first 6 months, but flattens markedly out at 1 and 2 years of age. At 2 years of age, the head circumference measured 37.5 cm (<3rd percentile), reflecting a progressive severe microcephaly, which was confirmed by MRI imaging (**Figure 1A**). Clinical symptoms in patient 1 became apparent immediately after birth with tonic-clonic seizures, relapsing vomiting, and feeding difficulties. In addition, a muscle hypertonus and a tetraspastic movement dysfunction was noted. During further infantile development, the girl did not achieve any major developmental milestones and never attained the ability to sit, stand, or walk. Throughout life, no voluntary movements of limbs or any parts of the body have been observed. A severe mental retardation was diagnosed from infantile development on with severe deficits in perception and an inability to establish any form of targeted contact with other individuals. In line with the majority of patients with ASNSD, a cortical blindness was diagnosed. Though, the hearing ability is not significantly impaired and acoustic stimulation is used as therapeutic measures of treatment. In addition, a cutis verticis gyrate was diagnosed around the age of 10 years.

Due to relapsing incidents of aspiration, a fundoplication and the insertion of a percutaneous endoscopic gastrostoma (PEG) were undertaken at the age of 4 years. She received an intrathecal pain-pump implantation at the age of 9 years in order to provide a constant supply with the muscle-relaxant drug baclofen. At the age of 16 years the girl demonstrated a profound psychomotor retardation with an apathetic behavior and a severe deficit in perception and no signs of improvement in comparison to the initial clinical picture during infancy. The patient showed a height of 135 cm (<3rd percentile), a weight of 49 kg (10th percentile), and a head circumference of 45 cm (<3rd percentile) at that age. She suffers from hebdomadal tonic-clonic epileptic seizures and frequent painful spasms of the upper extremities together with events of opisthotonus that last for a few minutes. The electro-encephalogram (EEG) demonstrates a severe, diffuse brain dysfunction in addition to the characteristic pattern of tonic-clonic epileptic seizures. At present, the medication comprises sulthiame, tizanidine, phenobarbital, and valproic acid. In addition, the girl receives constant physiotherapy and attends a social organization for blind, handicapped children during daytime.

The second patient is the sibling of patient 1 and was born 3 years after her sister (for a comparison of both patients see also **Figure 1B**). Patient 2 has a healthy twin brother. Both children were born in the 38th week of gestation via cesarean section after uncontrollable labor. The newborn girl presented with a birth weight of 2,450 g (<3rd percentile), a height of 45 cm (<3rd percentile), and a head circumference of 30 cm (<3rd percentile). Microcephaly had been diagnosed already during pregnancy via ultrasonography. Comparable to her sister, the relative increase in head circumference of patient 2 paralleled the standard curve during the first 6 months of age, but then flattened out with a head circumference of 37 cm (<3rd percentile) at the age of 2 years, reflecting a severe, progressive microcephaly. Overall, the clinical symptoms of patients 1 and 2 are nearly identical. In detail, patient 2 developed the first neurological symptoms at the age of 4 weeks with epileptic seizures, muscle hypertonus, and a progressive tetraspastic motor dysfunction. Like her sister, she suffers from a cortical blindness. A cutis verticis gyrate was also diagnosed during childhood. Due to gastroesophageal reflux and feeding problems the girl received a fundoplication and the insertion of a PEG at the age of 1 year. Again, no developmental milestones with regard to motor development were achieved, and the patient never developed the ability to carry out voluntary movements or axial control. She showed a severe psychomotor retardation and pronounced deficits in perception from early infancy on, which did not significantly improve during further development and parallels the clinical picture of her older sister. The girl is 124 cm tall (<3rd percentile), has a body weight of 43 kg (25th percentile), and a head circumference of 43 cm (<3rd percentile). The EEG demonstrates a severe diffuse brain dysfunction together with an overall slowing and flattening of the amplitudes. The girl suffers from relapsing tonic-clonic seizures in addition to a severe tetraspasticity. The medication comprises baclofen, sulthiame, gabapentin, and levetiracetam next to physical therapy. Like her sister, she is attending a social organization for blind, handicapped children. At time of submission, patient 1 was 19 years old and her sister, patient 2, 16 years of age. Both patients show a stable, overall unaltered health situation regarding the specific disease syndrome as well as with respect to the general health condition within the last years. Both patients live in their family at home.

## Laboratory Investigations

### Reference Sequence

As some discrepancies and inconsistencies exist about in the denomination of mutations in *ASNS* partly due to the use of different reference sequences (see results and **Table 1**), all positions in the ASNS gene are annotated to RefSeq NM_133436.3, Ensembl ENST00000394309.3, GRCh37 in this publication. Amino acid positions were assigned using NCBI NP_597680.2 and UniProtKB P082431 as reference. Whole exome sequence data were mapped and aligned to Human Genome Build GRCh37/hg19.

### Whole Exome Sequencing

A total of 50 ng of genomic DNA were used for paired-end libraries synthesis with the Nextera DNA Library Prep kit (Illumina) according to the instructions of the manufacturer. A pool of up to eight libraries was used for exomes enrichment and indexing with the Nextera Rapid Capture Expanded Exomes kit (Illumina). Cluster generation was performed with the library pool at a concentration of 10 nM using an Illumina cBot. Paired-end reads of 100 bp were sequenced with an IlluminaHighScan-SQ sequencer at the sequencing core facility of the Faculty of Medicine (University Leipzig) using version 3 chemistry and flowcell according to the instructions of the manufacturer. After base calling with Real-Time Analysis software 1.13 (Illumina) demultiplexing of raw reads, adapter trimming, and quality filtering was done accordingly (Stokowy et al., 2014). Resulting read pairs were mapped to the human genome (hg19) using the BWA aligner (Li and Durbin, 2010). Mapped reads were further processed for variant calling according to the Best Practices workflow (DePristo et al., 2011; Van der Auwera et al., 2013) suggested by the Broad Institute using GATK 3.4 tools (McKenna et al., 2010). Finally, genomic variants were annotated and filtered using wANNOVAR^1^, a web based tool for the functional annotation of genetic variants (Yang and Wang, 2015). Variant filtering was applied for rare Mendelian disease using the default parameter settings. Briefly, non-synonymous coding and splicing variants were considered candidates if their minor allele frequency (MAF) in the 1000 Genomes Project and gnomAD exome databases was ≤1% for a recessive mode of inheritance or ≤0.01% for a dominant inheritance pattern. In addition, the same MAF thresholds were applied to exclude variants that occurred in an in-house exome database of unaffected control individuals.

### Sanger Sequencing

To validate the mutations in the ASNS coding sequence found by Whole Exome Sequencing (WES), all *ASNS* exons were sequenced in all family members and three independent healthy control samples using Sanger sequencing on an ABI PRISM 3130 × l Genetic Analyzer (Thermo Fisher Scientific, Waltham, MA, United States).

### Protein Modeling

The ASNS protein was modeled using the I-Tasser online server (Zhang Lab^2^, University of Michigan; Zhang, 2009; Roy et al., 2012; Yang and Zhang, 2015). *ASNS*-wild-type, -M201Wfs^∗^28 and -E389Q FASTA sequences were loaded and modeled using the crystal structure from *E. coli* as template (PDB 1CT9:1; DOI: 10.2210/pdb1ct9/pdb). The best fitting model for each query (positive C-score, model 1 for each) was downloaded and visualized using PyMOL software (The PyMOL Molecular Graphics System, Version 2.0 Schrödinger, LLC^3^).

### Identification of Mutations in *ASNS*

Using WES, the coding sequences of the genome of the two affected girls, their unaffected brother as well as their healthy parents were analyzed with wANNOVARbbb^1^ (Yang and Wang, 2015). We assumed two models for the analysis: (1) a rare recessive Mendelian disease model and (2) a *de novo* rare dominant Mendelian disease model. Pedigree structure excluded an autosomal dominant model of inheritance as both parents are unaffected. Further, X-chromosomal dominant inheritance was excluded for the same reason, and X-chromosomal recessive inheritance was excluded because of the unaffected father. All genetic variants consistent with a *de novo* dominant disease model (variants not detected in parents or brother) could be excluded because only one of the affected sisters carried the variant.

The rare recessive Mendelian disease model in wANNOVAR assumes that in case of an autosomal inherited disease at least two deleterious alleles (compound heterozygous or homozygous) need to be present in a gene. A gene was considered as a candidate if mutations were detected in both affected girls. Of those, all genes except for *ASNS* could be excluded, because either (a) identical variants were detected in the unaffected brother; (b) the mother was homozygous for the variants; (c) the haplotype was derived from the mother; or (d) the variant was found ≥2× in the controls, or have been found homozygous in a high number in the gnomAD database. Within *ASNS*, which has previously been associated with congenital microcephaly (Ruzzo et al., 2013; Gupta et al., 2017), two new mutations were identified: c.1165G > C, p.E389Q and c.601delA, p.M201Wfs^∗^28 (**Table 2**; all positions in the ASNS gene are annotated to RefSeq NM_133436.3, Ensembl ENST00000394309.3, GRCh37). Segregation in the family revealed compound heterozygous genotypes in both patients and confirmed the autosomal-recessive mode of inheritance. The unaffected brother carried only one of the variants (**Figure 1C**).

**Table 2:** Sequencing result in cases and controls.

Location	Exon 1	Intron 1–2	Exon 5		Exon 10	
Variant	Ins A	Ins A	Del A (^∗^)	T > A	G > C (^∗^)	C > G
rs-number	rs58521276	rs796621224		rs1049674		rs1049677
Position in cDNA	5′ UTR	Intron	c.601delA	c.629T > A	c.1165G > C	c.1209C > G
Position in protein			p.M201Wfs^∗^28	p.V210E	p.E389Q	p.L403L
Functional consequence	UTR variant	Intron variant	Frameshift variant	Missense	Missense	Synonymous codon
Index patient	A/A	A/A	Del -/A	T/T	G/C	C/C
Sister	Ins A/Ins A	A/A	Del -/A	T/T	G/C	C/C
Brother	A/Ins A	A/Ins A	A/A	T/T	G/C	C/G
Father	A/Ins A	A/Ins A	Del -/A	T/T	G/G	C/G
Mother	Ins A/Ins A	A/Ins A	A/A	T/T	G/C	C/C
Control 1	Ins A/Ins A	A/A	A/A	A/T	G/G	C/G
Control 2	A/A	A/A	A/A	A/T	G/G	C/G
Control 3	A/Ins A	A/A	A/A	A/T	G/G	C/G
Prediction-Tools						
Polyphen	n.d.	n.d.	n.d.	0	0.99	n.d.
SIFT	n.d.	n.d.	n.d.	0.4	0.02	1
SNP&GO	n.d.	n.d.	n.d.	0.347	0.890	n.d.
MutPred	n.d.	n.d.	n.d.	0.22	0.67	n.d.

*(^∗^) Mutations found in the family. UTR, untranslated region; Ins, insertion; Del, deletion; n.d., not determined/no prediction possible. Polyphen (http://genetics.bwh.harvard.edu/pph2/): 0−0.452 benign; 0.453−0.955 possibly damaging; 0.956−1 probably damaging. SIFT (http://sift.jcvi.org/): value ≤0.05 damaging, value ≥0.05 benign. SNP&GO (http://snps-and-go.biocomp.unibo.it/snps-and-go/): value ≥0.5 most likely damaging. MutPred (http://mutpred.mutdb.org/): value = probability of a damaging mutation. Reference Sequence NM_133436.3.*

The WES data were validated by Sanger Sequencing. The mutations c.601delA and c.1165G > C co-segregated with the disease in the family and were not found in population matched controls (**Figure 1C** and **Table 2**). In addition, four further known polymorphisms were detected that did not co-segregate with the disease. Two are located in the 5′ UTR or the intron respectively (rs58521276 [Exon 1], rs796621224 [Intron 1-2]), one in exon 5 (rs1049674), and one in exon 10 (rs1049677). The newly identified mutations were not found in dbSNP, Exome Sequencing Project (ESP), Exome Aggregation Consortium (ExAC), Human Gene Mutation Database (HGMD; public version), and 1000 Genomes databases, suggesting that these variants are very rare in the population. The novel genetic variants in ASNS described in this work have been submitted to the ClinVar database (c.601delA: accession number: SCV000778369; c.1165G > C: accession number: SCV000778370).

### Prediction of Functional Consequences and Protein Modeling

The two novel mutations were inherited by the patient’s family in a compound heterozygous manner (**Figure 1C**). Mutation c.1165G > C [p.E389Q] exchanges glutamate by glutamine at position 389 of the protein (**Figure 1C**), which was predicted as a most likely damaging mutation by all prediction tools used (**Table 2**). Mutation c.601delA [p.M201Wfs^∗^28] results in a frame shift and induces a new stop codon after amino acid 227 thereby deleting more than half of the protein (total wild-type protein size 561 aa). While both new variants are not recorded yet in the public databases (see above), a variant different from the c.1165G > C mutation is described in Ensembl and dbSNP at the same *ASNS* coding sequence position (rs948326794 c.1165G > T, MAF < 0.01) leading to a premature stop codon. This variant was submitted by TOPMED (Goncalo Abecasis, Center for Statistical Genetics, Biostatistics Department, Ann Arbor, MI, United States). No further information on this variant is currently available in the literature and – to our knowledge – no cases of microcephaly carrying this variant have been reported so far.

Modeling of the 3D structure of ASNS was performed to localize the position of the mutated amino acids and the impact of the mutation on the protein structure. As no structure of the human ASNS has been reported so far, the model was based on the structure of *E. coli* ASNS (**Figure 1D**; accession number 1CT9 in the RCSB PDB database) as described previously (Gupta et al., 2017; Lomelino et al., 2017; Sacharow et al., 2017; Yamamoto et al., 2017). The ASNS enzyme consists of two protein domains, the glutamine amidotransferase domain (aa 2 to 191; UniProtKB Entry P082431; *orange* in **Figures 1D–G**) and the ASNS domain (aa 213 to 536; *green* in **Figures 1D–G**). Glutamate at position E389 is located in an alpha-helix within the ASNS domain rather at the surface of the enzyme (**Figure 1E**). Mutation E389Q might destabilize ASNS (**Figure 1G** and data not shown) as predicted by DUET (protein stability upon mutation; Pires et al., 2014), INPS-MD (Impact of Non-synonymous mutations on Protein Stability – Multi-Dimension; Fariselli et al., 2015), and MUPro (Prediction of Protein Stability Changes for Single Site Mutations from Sequences; Cheng et al., 2006). As the other mutation identified – c.601delA [p.M201Wfs^∗^28] – preserves the glutamine amidotransferase domain while the ASNS domain is almost completely lacking (**Figure 1F**) it is not surprising that this protein is predicted to be non-functional as an ASNS enzyme anymore.

### Variants in *ASNS* Associated With Asparagine Synthetase Deficiency

Including the novel mutations described here, 26 mutations are known in *ASNS* so far likely causing ASNSD which results in a clinical picture of microcephaly (**Table 1**; 23 mutations reported in scientific journals, one [#3 in **Table 1**] as a poster abstract, two mutations [#20, #23 in **Table 1**] were self-reported on websites). In several publications reporting *ASNS* mutations in patients, different reference sequences were used and some mutations were reported inconsistently even within one publication. For the overview provided in **Table 1** all mutations have been mapped to the reference sequence NM_133436.3, revealing that two mutations previously reported as different mutations are in fact the same variant (at position c.1193, #17 in **Table 1**) as also suggested recently (Lomelino et al., 2017).

**FIGURE 2:**
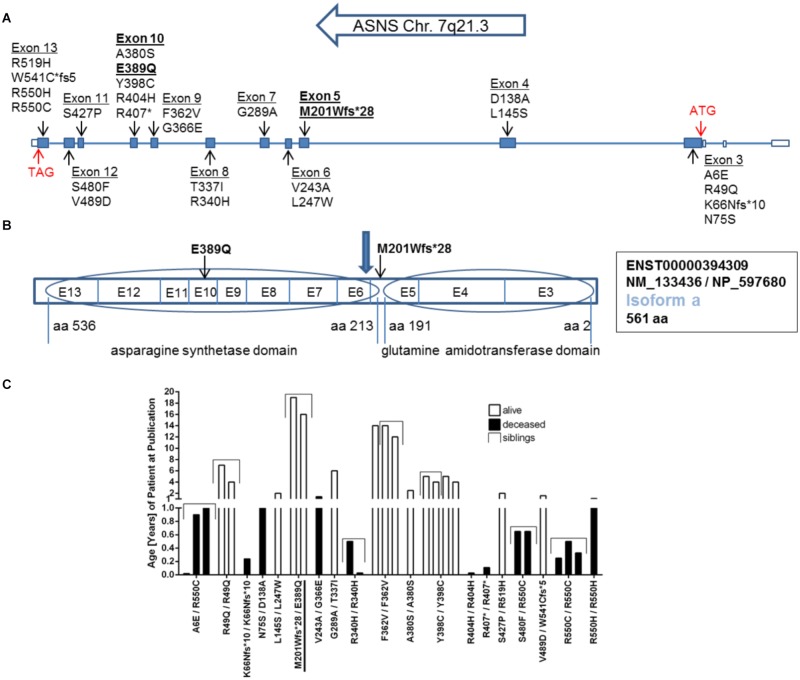
Mutations in the *ASNS* gene. **(A)** Schematic representation of the gene structure of ASNS including the location of mutations so far described for ASNS deficiency in the literature and the newly identified mutations in *bold*. *Filled boxes* indicate translated exons. The translation start site (ATG) is located in exon 3, the stop codon (TAG) in exon 13 (*red arrows*) referring to transcripts for isoform a. **(B)** Exons coding for the glutamine amidotransferase domain and the asparagine synthetase domain in the protein. The mutation M201Wfs^∗^28 is located between both domains and the *blue arrow* indicates the position of the stop codon generated by the M201Wfs^∗^28 mutation. The mutation E389Q is located in exon 10. E-exon, aa-amino acid. **(C)** Survival of children born alive and with confirmed homozygous or compound heterozygous mutations in *ASNS.* Given is the age in years as reported in the patient description at the time point of the initial publication or the follow up study. The compound heterozygous mutation M201Wfs^∗^28/E389Q presented here is *underlined*. See **Table 1** for references regarding the other mutations.

Within *ASNS*, these mutations are scattered throughout the coding sequence of the glutaminase and the ASNS domains, but also affecting amino acids between the two domains and the very C-terminal amino acids in the protein (**Figures 2A,B**). All mutated amino acids are highly conserved among different species from zebrafish to humans (Supplementary Figure 1). Strikingly, while in zebrafish E389 is replaced by aspartate (Supplementary Figure 1), the glutamate at this position is also present in *Drosophila melanogaster* (NP_993132.1), *Saccharomyces cerevisiae* (NP_011640.1), and even *E. coli* (WP_000337071.1; data not shown).

The clinical data of many of the patients with ASNSD reported so far has recently been summarized (Gupta et al., 2017). An obvious heterogeneity between all these patients is the different lethality and life span (**Figure 2C**). This analysis has to be considered with caution as other factors besides the direct consequence of the *ASNS* mutations can affect the life span in addition to the *ASNS* mutation. Furthermore, several patients were still alive at the time of publication so their life span is unknown. Nevertheless, some mutations appear to be associated with shorter life span, i.e., higher severity of the disease (e.g., R550C; **Figure 2C**).

## Discussion

Mutations in *ASNS* have recently been identified as the cause of microcephaly and severe defects in brain development in human patients (Ruzzo et al., 2013). In a family with two girls with microcephaly, we have identified two novel mutations. These mutations have not been reported in the current human genome databases (dbSNP, ESP, ExAC, HGMD; public version, 1000 Genomes, and gnomAD) suggesting that these are very rare genetic variants like previously reported disease causing mutations in ASNS (**Table 1**). While some of these mutations are included in these databases and reported as singletons or with minor allele frequencies <1/10,000, respectively (all heterozygous) in the general population (data not shown), around half of the *ASNS* mutations described to be associated with microcephaly are reported as private so far which includes also the variants identified here.

While the mutations in ASNS have been unequivocally identified as the cause of the disease condition, the pathophysiology remains enigmatic. Two aspects need to be considered in this context: (1) What is the effect of the mutation on the ASNS protein? (2) Why does a (assumed) loss in ASNS activity result in the phenotype observed in the patients?

Firstly, it has been suggested that mutations in ASNS lead to reduced expression (Ruzzo et al., 2013), and/or to a reduced stability of the protein (Yamamoto et al., 2017). The E389Q mutation is located in the second helix of a helix-loop-helix motif and the A380S mutation in the first helix of this motive destabilizes the ASNS protein (Gupta et al., 2017). Consistently, prediction tools suggest a destabilization of the ASNS-E389Q protein. E389 is conserved throughout evolution from *E. coli* to humans, with the exception of *Danio rerio* where it is replaced by aspartate, indicating that the negative charge of the amino acid at this position is important for ASNS function. E389 is localized rather on the surface of the ASNS protein near the binding pocket of ATP and aspartate (Gupta et al., 2017); however, whether and how E389 contributes to the binding of these molecules remains to be addressed by further investigations. Furthermore, mutations introducing a premature stop codon, like the c.601delA [p.M201Wfs^∗^28] mutation reported here, often result in expression of non-functional truncated proteins, which may be cleared by lysosomal degradation immediately after translation. Alternatively, nonsense mediated decay of mutant mRNA might result in fast degradation of the respective mRNAs (Miller and Pearce, 2014; Ottens and Gehring, 2016). However, while no measurements of the specific activity of the different ASNS mutants have been reported so far, it is very likely that – by whatever mechanism – the observed *ASNS* mutations finally result in a reduced or absent enzymatic activity of ASNS. This is consistent with the impaired growth of fibroblasts in the absence of Asn as reported for the G289A/T337I compound heterozygous mutation (Palmer et al., 2015).

Secondly, how does a reduced activity of ASNS result in microcephaly? ASNS synthesizes Asn and Glu from Asp and Gln; and it has been suggested that loss of ASNS activity might result in a decrease in Asn availability in the brain (Ruzzo et al., 2013). However, Asn is generally considered as a non-essential amino acid and can be provided to the developing fetus in sufficient amounts to enable normal development of peripheral organs. While no data is available on the permeability of Asn through the blood–brain barrier in the developing human fetus, it has therefore been suggested that lack of ASNS activity disturbs an intricate metabolic balance of amino acids including Asn, Asp, Glu, and Gln resulting in disturbed brain development and function (Sacharow et al., 2017). Interestingly, other deficiencies of enzymes involved in the synthesis of non-essential amino acids including serine, glutamine, and proline have been reported, which all show a severe neurological and/or neurodevelopmental phenotype (de Koning, 2017). With the exception of skin deficiencies reported for several cases, these neurological symptoms appear to be a rather isolated phenotype also in these conditions similar to ASNSD (de Koning, 2017). Therefore, as “it is puzzling how a ubiquitously expressed enzyme defect can affect the central nervous system in such a specific way” (de Koning, 2017), the pathomechanisms of these mutations of enzymes of amino acid biosynthesis are still enigmatic and deserve further investigations.

In conclusion, we here present two novel mutations in *ASNS* identified in a family with two children with microcephaly. These add to the recently rapid growing list of reported mutations in *ASNS* in children with this condition. However, while identification of mutations will help to diagnose patients, it will still be a long way to unravel the underlying pathophysiology or even to develop treatment for the patients.

## Author Contributions

DS, AS, PGH, UW, SK, JB, and KK: acquisition of data. DS, AS, RSt, JK, UW, SK, JTH, RSc, KK, KH, PK, and JH: analysis and interpretation of data. PK and JH: conception of the project. KH, PK, and JH: supervised the project. DS, RSt, PK, and JH with input of all coauthors: wrote the paper. All authors approved the final version of the MS.

## Conflict of Interest Statement

The authors declare that the research was conducted in the absence of any commercial or financial relationships that could be construed as a potential conflict of interest.

## Footnotes

1 http://wannovar.wglab.org

2 https://zhanglab.ccmb.med.umich.edu/I-TASSER/

3 https://pymol.org/2/
