# Supplementary material for: Novel Mutations in the Asparagine Synthetase Gene (ASNS) Associated With Microcephaly
Source: Front Genet. 2018 Jul 13;9:245. doi: 10.3389/fgene.2018.00245 (PMC6053511; doi:10.3389/fgene.2018.00245)
Supplement: Supplementary file 1 [file Image_1.PDF]

Supplementary Material

Novel mutations in the asparagine synthetase gene (*ASNS*) associated with microcephaly

Dorit Schleinitz, Anna Seidel, Ruth Stassart, Jürgen Klammt, Petra G. Hirrlinger, Ulrike Winkler, Susanne Köhler, John T. Heiker, Ria Schönauer, Joanna Bialek, Knut Krohn, Katrin Hoffmann, Peter Kovacs*, Johannes Hirrlinger*

*** Correspondence:** peter.kovacs@medizin.uni-leipzig.de
johannnes.hirrlinger@medizin.uni-leipzig.de

Supplementary Figure 1. Conservation of amino acids in ASNS. Sequences were derived from the UniProt database (UniPritKB; Pundir et al., 2017; The UniProt consortium, 2017) and aligned using the Clustal Omega (1.2.4) multiple sequence alignment program implemented in UniProt. The following organisms were included: Human (*Homo sapiens*), Sumatran orangutan (*Pongo abelii*), Rhesus macaque (*Macaca mulatta*), Bovine (*Bos taurus*), Chicken (*Gallus gallus*), Mouse (*Mus musculus*), Rat (*Rattus norvegicus*), Chinese hamster (*Cricetulus griseus*), and Zebrafish (*Danio rerio*). Conservation: *(asterix) single fully conserved residue; :(colon) conservation between groups of strongly similar properties – scoring >0.5 in the Gonnet PAM 250 matrix; .(period) conservation between groups of weakly similar properties – scoring =<0.5 in the Gonnet PAM 250 matrix (according to Uniprot; http://www.uniprot.org). Mutations described for ASNSD are given in blue, the mutations found in the family of this case report in green.

References

Pundir, S., Martin, M. J., & O'Donovan, C. (2017). UniProt Protein Knowledgebase. Methods Mol Biol, 1558, 41-55.

The UniProt consortium. (2017). UniProt: the universal protein knowledgebase. Nucleic Acids Res, 45, D158-D169.

```
UniProtKB ASNS              A6E                                            R49Q
P08243|HUMAN           MCGIWALFGSDDCLSVQCLSAMKIAHRGPDAFRFENVNGYTNCCFGFHRLAVVDPLFGMQ     60
Q5R6W9|ORANGUTAN       MCGIWALFGSDDCLSVQCLSAMKIAHRGPDAFRFENVNGYTNCCFGFHRLAVVDPLFGMQ     60
F6SSI3|MACAQUE         MCGIWALFGSDDCLSVQCLSAMKIAHRGPDAFRFENVNGYTNCCFGFHRLAVVDPLFGMQ     60
Q1LZA3|BOVINE          MCGIWALFGSDDCLSVQCLSAMKIAHRGPDAFRFENVNGYTNCCFGFHRLAVVDQLFGMQ     60
Q5ZJU3|CHICKEN         MCGIWALFGSDECLSVQCLSAMKIAHRGPDAFRFENVNGFTNCCFGFHRLAVVDQLYGMQ     60
Q61024|MOUSE           MCGIWALFGSDDCLSVQCLSAMKIAHRGPDAFRFENVNGYTNCCFGFHRLAVVDPLFGMQ     60
P49088|RAT             MCGIWALFGSDDCLSVQCLSAMKIAHRGPDAFRFENVNGYTNCCFGFHRLAVVDPLFGMQ     60
P19891|HAMSTER         MCGIWALFGSDDCLSVQCLSAMKIAHRGPDAFRFENVNGYTNCCFGFHRLAVVDPLFGMQ     60
A3KQK2|ZEBRAFISH       MCGIWALFGSDECLSVQCTNAMKIFHRGPDAFRFENVNGFTNCCFGFHRLAIVDQLYGMQ     60
Conservation    1      ***********:****** .**** **************:***********:** *:***     60
                        ↪glutamine amidotransferase domain
                            K66Nfs*10 N75S
P08243|HUMAN           PIRVKKYPYLWLCYNGEIYNHKKMQQHFEFEYQTKVDGEIILHLYDKGGIEQTICMLDGV     120
Q5R6W9|ORANGUTAN       PIRVKKYPYLWLCYNGEIYNHKKMQQHFEFEYQTKVDGEIILHLYDKGGIEQTICMLDGV     120
F6SSI3|MACAQUE         PIRVKNYPYLWLCYNGEIYNYKKMQRHFEFEYQTNVDGEIILHLYDKGGIEQTICMLDGV     120
Q1LZA3|BOVINE          PIRVKKYPYLWLCYNGEIYNHKKLQHHFEFEYQTKVDGEIILHLYDKGGIEQTVCMLDGV     120
Q5ZJU3|CHICKEN         PIRVKKFPYLWLCYNGEIYNFKQLQEQFGFEYQTLVDGEVILHLYNRGGIEQTASMLDGV     120
Q61024|MOUSE           PIRVRKYPYLWLCYNGEIYNHKALQQRFEFEYQTNVDGEIILHLYDKGGIEKTICMLDGV     120
P49088|RAT             PIRVRKYPYLWLCYNGEIYNHKALQQRFEFEYQTNVDGEIILHLYDKGGIEKTICMLDGV     120
P19891|HAMSTER         PIRVKKYPYLWLCYNGEIYNHKALQQRFEFEYQTNVDGEIILHLYDKGGIEQTICMLDGV     120
A3KQK2|ZEBRAFISH       PLRVKKFPYLWLCYNGEIYNHIKLKNHFEFDYQTKVDGEILLHLYDRFGIEKMCSLLDGV     120
Conservation    61     *:**:.:*************.  ::.:* *:*** ****::****:: ***:  .:****     120
                                         D138A  L145S
P08243|HUMAN           FAFVLLDTANKKVFLGRDTYGVRPLFKAMTEDGFLAVCSEAKGLVTLKHSATPFLKVEPF     180
Q5R6W9|ORANGUTAN       FAFVLLDTATKKVFLGRDTYGVRPLFKAMTEDGFLAVCSEAKGLVTLKHSTTPFLKVEPF     180
F6SSI3|MACAQUE         FAFVLLDTANKKVFLGRDTYGVRPLFKAITEDGFLAVCSEAKGLVTLKHSTTPFLKVEPF     180
Q1LZA3|BOVINE          FAFILLDTANKKVFLGRDTYGVRPLFKAMTEDGFLAVCSEAKGLVNLKHSMTPFLKVEPF     180
Q5ZJU3|CHICKEN         FAFILLDTANRKVFLARDTYGVRPLFKVLTDDGFLGVCSEAKGLINLKHSTSLFPKVEPF     180
Q61024|MOUSE           FAFILLDTANKKVFLGRDTYGVRPLFKAMTEDGFLAVCSEAKGLVSLKHSTTPFLKVEPF     180
P49088|RAT             FAFILLDTANKKVFLGRDTYGVRPLFKALTEDGFLAVCSEAKGLVSLKHSTTPFLKVEPF     180
P19891|HAMSTER         FAFILLDTANKKVFLGRDTYGVRPLFKAMTEDGFLAVCSEAKGLVSLKHSTTPFLKVEPF     180
A3KQK2|ZEBRAFISH       FAFILLDTANRKVHLGRDTYGVRPLFRMLTDDGFLAVCSEGKGLTQIKHSM-PTAKITAF     179
Conservation    121    ***:*****.:**.*.**********: :*:****.****.***   :***      *:  *     180
                                                M201Wfs*28
P08243|HUMAN           LPGHYEVLDLKPNGKVASVEMVKYHHCRDVPLHALYDNVEKLFPGFEIETVKNNLRILFN     240
Q5R6W9|ORANGUTAN       LPGHYEVLDLKPNGKVASVEMVKYHHCRDEPLHALYDNVEKLFPGFEIETVKNNLRILFN     240
F6SSI3|MACAQUE         LPGHYEVLDLKPNGKVASVEMVKYHHCRDEPLHALYDNVDKLFPGFEIETVKNNLRILFN     240
Q1LZA3|BOVINE          LPGHYEVLDLKPNGKVASVEMVKHHHCRDEPLHALYDGVEKLFPGFEIETVKSNLRILFD     240
Q5ZJU3|CHICKEN         LPGHYEVLDLKPSGKVVSVEVVKFHSYKDEPLHAACDTVGNLPSGFDLETVKSNIRVLFE     240
Q61024|MOUSE           LPGHYEVLDLKPNGKVASVEMVKYHHCTDEPLHAIYDSVEKLFPGFDLETVKNNLRILFD     240
P49088|RAT             LPGHYEVLDLKPNGKVASVEMVKYHHCTDEPLHAIYDSVEKLFPGFEIETVKNNLRILFN     240
P19891|HAMSTER         LPGHYEVLDLKPNGKVASVEMVKYHHCRDEPLHALYDSVEKLFQGFELETVKSNLRILFD     240
A3KQK2|ZEBRAFISH       PPGHFEVFDLKLNGKVESVQMDRFHCCTDKPKHADFNKLEGLGTDFELETVKSNIRILFE     239
Conservation    181     ***:**:*** .*** **:: :.*   * * **  : :  *  .*::****.*:*:**:     240
                          end↩                          ↪asparagine synthetase domain
                       V243A L247W                                     G289A
P08243|HUMAN           NAVKKRLMTDRRIGCLLSGGLDSSLVAATLLKQLKEAQVQYPLQTFAIGMEDSPDLLAAR     300
Q5R6W9|ORANGUTAN       NAVKKRLMTDRRIGCLLSGGLDSSLVAATLLKQLKEARVQYPLQTFAIGMEDSPDLLAAR     300
F6SSI3|MACAQUE         NAVKKRLMTDRRIGCLLSGGLDSSLVAATLLKQLKEAQVQYPLQTFAIGMEDSPDLLAAR     300
Q1LZA3|BOVINE          NAVKKRLMTDRRIGCLLSGGLDSSLVAATLLKQLKEAQVQYPLQTFAIGMEDSPDLLAAR     300
Q5ZJU3|CHICKEN         NAVRKRLMAHRRIGCLLSGGLDSSLVAAVLLKLMKEMNIKYPLQTFAIGMENSPDLLAAR     300
Q61024|MOUSE           NAIKKRLMTDRRIGCLLSGGLDSSLVAASLLKQLKEAQVQYPLQTFAIGMEDSPDLLAAR     300
P49088|RAT             NAIKKRLMTDRRIGCLLSGGLDSSLVAASLLKQLKEAQVPYALQTFAIGMEDSPDLLAAR     300
P19891|HAMSTER         SAVRKRLMTDRRIGCLLSGGLDSSLVAASLLKQLKEAQVQYPLQTFAIGMEDSPDLLAAR     300
A3KQK2|ZEBRAFISH       DAVRKRLMAHRRIGCLLSGGLDSSLVAALLVKLAKEEKLPYPIQTFSIGAEDSPDVAAAR     299
Conservation    241    .*::****:.****************** *:*  ** .: * :***:** *:***: ***     300
                                                         T337I  R340H
P08243|HUMAN           KVADHIGSEHYEVLFNSEEGIQALDEVIFSLETYDITTVRASVGMYLISKYIRKNTDSVV     360
Q5R6W9|ORANGUTAN       KVADHIGSEHYEVLFNSEEGIQALDEVIFSLETYDITTVRASVGMYLISKYIRKNTDSVV     360
F6SSI3|MACAQUE         KVANHIGSEHYEVLFNSEEGIQALDEVIFSLETYDITTVRASVGMYLISKYIRKNTDNVV     360
Q1LZA3|BOVINE          KVANHIGSEHHEVLFNSEEGIQVLDEVIFSLETYDITTVRASVGMYLISKYIRKNTDSVV     360
Q5ZJU3|CHICKEN         KVAAHIGSEHHEVIFNSEEGIQAVEEVIFSLETYDITTVRASIGMYLVSKYIRKKTDSVV     360
Q61024|MOUSE           KVANYIGSEHHEVLFNSEEGIQALDEVIFSLETYDITTVRASVGMYLISKYIRKNTDSVV     360
P49088|RAT             KVANYIGSEHHEVLFNSEEGIQSLDEVIFSLETYDITTVRASVGMYLISKYIRKNTDSVV     360
P19891|HAMSTER         KVANYIGSEHHEVLFNSEEGIQALDEVIFSLETYDITTVRASVGMYLISKYIRKNTDSVV     360
A3KQK2|ZEBRAFISH       KVADYIGSEHHVVNFTPEEGISALEDVIVHLESYDITTVRASIGMYLVSKYIREKTDSVV     359
Conservation    301    *** :*****: * *. ****. ::**. **:***********:****:*****::**.**     360
                       F362V G366E      A380S   E389Q  Y398C R404H R407*
P08243|HUMAN           IFSGEGSDELTQGYIYFHKAPSPEKAEEESERLLRELYLFDVLRADRTTAAHGLELRVPF     420
Q5R6W9|ORANGUTAN       IFSGEGSDELTQGYIYFHKAPSPEKAEEESERLLRELYLFDVLRADRTTAAHGLELRVPF     420
F6SSI3|MACAQUE         IFSGEGSDELTQGYIYFHKAPSPEEAEEESERLLRELYLFDVLRADRTTAAHGLELRVPF     420
Q1LZA3|BOVINE          IFSGEGSDELTQGYIYFHKAPSPEKAEEESERLLRELYLFDVLRADRTTAAHGLELRVPF     420
Q5ZJU3|CHICKEN         IFSGEGSDELTQGYIYFHKAPSPEEAAEESERLLKELYLFDVLRADRTTAAHGLELRVPF     420
Q61024|MOUSE           IFSGEGSDELTQGYIYFHKAPSPEKAEEESERLLKELYLFDVLRADRTTAAHGLELRVPF     420
P49088|RAT             IFSGEGSDELTQGYIYFHKAPSPEKAEEESERLLKELYLFDVLRADRTTAAHGLELRVPF     420
P19891|HAMSTER         IFSGEGSDELTQGYIYFHKAPSPEKAEEESERLLKELYLFDVLRADRTTAAHGLELRVPF     420
A3KQK2|ZEBRAFISH       IFSGEGSDELTQGYIYFHKAPSPKAGAEDSVRLLEELYLFDVLRADRTTAAHGLELRVPF     419
Conservation    361    ************************: . *:* ***.**************************     420
                             S427P                                                  S480F
P08243|HUMAN           LDHRFSSYYLSLPPEMRIPKNGIEKHLLRETFEDSNLIPKEILWRPKEAFSDGITSVKNS     480
Q5R6W9|ORANGUTAN       LDHRFSSYYLSLPPEMRIPKNGIEKHLLRETFEDSNLIPKEILWRPKEAFSDGITSVKNS     480
F6SSI3|MACAQUE         LDHRFSSYYLSLPPEMRTPKNGIEKHLLRETFEESNLIPKEILWRPKEAFSDGITSVKNS     480
Q1LZA3|BOVINE          LDHRFSSYYLSLPPDMRVPKNGIEKHLLRETFEDSNLIPKEILWRPKEAFSDGITSVKNS     480
Q5ZJU3|CHICKEN         LDHRFTSYYLSLPAELRIPKNGIEKYLLRQSFEDSNLLPKEILWRPKEAFSDGIASVKKS     480
Q61024|MOUSE           LDHRFSSYYLSLPPDMRIPKNGIEKHLLRETFEDCNLLPKEILWRPKEAFSDGITSVKNS     480
P49088|RAT             LDHRFSSYYLSLPPEMRIPKDGIEKHLLRETFEDSNLLPKEILWRPKEAFSDGITSVKNS     480
P19891|HAMSTER         LDHRFSSYYLSLPPEMRIPKNGIEKHLLRETFEDSNLLPKEILWRPKEAFSDGITSVKNS     480
A3KQK2|ZEBRAFISH       LDHRFTAYYLSLPEEMRVPKNGVEKHLLRDAFDGMNLIPDEILWRRKEAFSDGLTSVKKS     479
Conservation    421    *****:.****** ::* **:*:**:***::*:  **:*.***** *******::***:*     480
                              V489D                               R519H
P08243|HUMAN           WFKILQEYVEHQVDDAMMANAAQKFPFNTPKTKEGYYYRQVFERHYPGRADWLSHYWMPK     540
Q5R6W9|ORANGUTAN       WFKILQEYVEHQVDDAMMANAAQKFPFNTPKTKEGYYYRQVFERHYPGRADWLSHYWMPK     540
F6SSI3|MACAQUE         WFKILQEYVEHQVDDAMMANAAQKFPFNTPKTKEGYYYRQIFERHYPGRADWLSHYWMPK     540
Q1LZA3|BOVINE          WFRILQDYIEHQVDDAAMASAAQKFPINTPKTKEGYYYRQIFENHYPGRADWLPHYWMPR     540
Q5ZJU3|CHICKEN         WFSILQDYIDQQVDDLLLEKAAEKYPFNPPRTKESYYYRQIFEKHYPGRSSWLPHYWMPR     540
Q61024|MOUSE           WFKILQDYVEHQVDDEMMSAASQKFPFNTPKTKEGYFYRQIFERHYPGRADWLTHYWMPK     540
P49088|RAT             WFKILQDFVEHQVDDAMMSEASQKFPFNTPQTKEGYYYRQIFEHHYPGRADWLTHYWMPK     540
P19891|HAMSTER         WFKILQDYVEHQVDDEMMATAAQKFPFNTPKTKEGYYYRQIFERHYPGRADWLTHYWMPK     540
A3KQK2|ZEBRAFISH       WYTSLQEHIESELNDSQLENANKLYHINPPTTKEGLFIRQIFEKHYPGQGEWTPHYWMPR     539
Conservation    481    *:  **:.:: :::*  :  * : : :* * ***. : **:**.****:..*  *****:     540
                                                                        end↩
                      W541Cfs*5 R550C/H
P08243|HUMAN           WINATDPSARTLTHYKSAVKA          561
Q5R6W9|ORANGUTAN       WINATDPSARTLTHYKSAVKA          561
F6SSI3|MACAQUE         WINATDPSARTLTHYKSAAKA          561
Q1LZA3|BOVINE          WTNATDPSARTLTHYKAAAKA          561
Q5ZJU3|CHICKEN         WVEATDPSARTLKHYKSAIQE          561
Q61024|MOUSE           WINATDPSARTLTHYKSAAKA          561
P49088|RAT             WINATDPSARTLTHYKSTAKA          561
P19891|HAMSTER         WINATDPSARTLTHYKSAAKA          561
A3KQK2|ZEBRAFISH       WIKATDPSARTLSIYKPDKEQ          560
Conservation    541    * :*********. **   :
```
